# Continuous murmur - the auscultatory expression of a variety of pathological conditions

**Published:** 2012-03-05

**Authors:** C Ginghină, OA Năstase, I Ghiorghiu, L Egher

**Affiliations:** * “Carol Davila” University of Medicine and Pharmacy, Bucharest; ** Cardiology Department, “Prof Dr CC Iliescu” Emergency Institute of Cardiovascular Diseases Bucharest

**Keywords:** continuous murmur, heart auscultation, diagnosis, incidence, congenital heart disease

## Abstract

Continuous murmur is a peculiarity of cardiovascular auscultation, relatively rare, which often hides complex cardiovascular diseases. This article is a review of literature data related to the continuous murmurs accompanied by commenting and illustrating them through our own cases.

Recognizing of a continuous murmur and understanding the cardiovascular pathologies that it can hide, is a challenge in current practice.

Cardiac auscultation is a fundamental step in diagnosing heart disease. This requires: a good technique and experience, as Froment R. said "auscultation (of the heart) is a music; which supposed a very long sensory education" [**11**].

Knowing the conditions that determinate continuous murmur, can facilitate recognition of this type of murmur and then establish the diagnosis [**2,4,6**].

**Definition.** *The continuous murmur is the murmur that begins in systole and continues without interruption, encompassing the second sound, throughout diastole or part of thereof [**2**].The murmur that disappears completely before the next first sound, is still considered continuous, if the systolic part of it continues without interruption during the second sound [**2,4,6**].*

The continuous murmurs are often intense, rough and accompanied by quivering [**7**].

Depending on the location of the continuous murmur and characteristics of its components, a differential diagnosis can be made; for example continuous murmurs of patent ductus arteriosus is the loudest left infraclavicular with a more pronounced systolic component, whereas a coronary artery fistula can be heard anywhere in the heart area (depending on the origin and drainage) and diastolic component in this case are loudest.

The presence of a systolic-diastolic murmur (systolic murmur and diastolic murmur) so called a to-and-fro murmur, is not a continuous murmur, being different, by separating the two murmurs through a small "silence". A to-and-fro murmur, involves two components: a systolic one, in which the blood flows in one direction, and a diastolic one in which the blood flows in the opposite direction, while those with true continuous murmur, the blood flows in the same direction in both systole and diastole [**4**]. 

Double murmur occurs most frequently in cases in which there is a valvular stenosis and a valvular regurgitation (aortic, mitral, tricuspid or pulmonary). This is due to a major reshuffle of valve cusps, they cannot open or close, being fenestrated, shortened or immobilized [**7**].

## Etiology

Continuous murmurs are generated by a continuous blood flow shunting from the high-pressure or high resistance circulation to the low-pressure or low resistance circulation, throughout systole and diastole.

**Figure 1:**
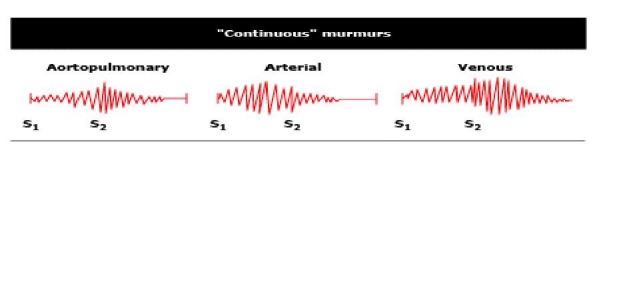
In this image the characteristics of a continuous murmur (modified after [**2**]) are represented. − continuous murmur from aortopulmonary communications- it is loudest around the S2. − arterial continuous murmur –it is characterized by a more pronounced systolic component. − venous continuous murmur- it is characterized by a more pronounced diastolic component. Legend figure 1: S1-first sound; S2-second sound.

This murmur (**Figure 1**) is mainly due to:

 - aortic-pulmonary communications,

 - arteriovenous communications (fistulas),

 - changes in arterial flows,

 - changes in veins flows [**2**].

## Classification

**Continuous murmurs** can be:

− **physiological**- murmurs occurring only under certain conditions without any structural abnormality at that level (mammary soufflé heard during pregnancy or cervical venous hum most commonly heard in sitting position, in the supraclavicular fossa, in healthy children). 

− **pathological**- secondary of vascular changes that allow the passage of blood from a high pressure area into a low pressure area, or increased resistance in the arterial circulation by a severe fixed arterial stenosis [**1**]. These pathologies may be secondary to an acquired trauma, spontaneous (rupture of an aneurysm in venous system), iatrogenic (after interventional or surgical procedures) or may be simply congenital defects.

A physiological classification of continuous murmurs as described by Myers and coworkers (**Table 1**).

**Table 1:** Physiologic classification of continuous murmurs (according to [**3**]).

Continuous murmurs caused by rapid blood flow :
Venous hum
Mammary soufflé
Hemangioma
Hyperthyroidism
Acute alcoholic hepatitis
Hyperemia of neoplasm (hepatoma, renal cell carcinoma, Paget disease)
Continuous murmurs caused by high- to low-pressure shunts :
Systemic artery to pulmonary artery (patent ductus arteriosus, aortopulmonary window, truncus arteriosus, pulmonary atresia, anomalous left coronary, bronchiectasis, sequestration of the lung)
Systemic artery to right heart (ruptured sinus of Valsalva, coronary artery fistula)
Left-to-right atrial shunting (Lutembacher syndrome, mitral atresia plus atrial septal defect)
Venovenous shunts (anomalous pulmonary veins, portosystemic shunts)
Arteriovenous fistula (systemic or pulmonary)
Continuous murmurs secondary to localized arterial sever stenosis:
Coarctation of the aorta
Branch pulmonary stenosis
Carotid stenosis
Celiac mesenteric stenosis
Renal stenosis
Femoral stenosis
Coronary stenosis

## Clinic

For a good presentation of the clinical context in which continuous murmurs can appear, they can be classified according to the place of auscultation (**Table 2**).

**Table 2:** Classification of continuous murmur after them sites of auscultation

		Patent ductus arteriosus
		Coronary arteriovenous fistulas
	Precordial	Sinus of Valsalva aneurysm ruptured into right cavities
THORACIC		Aortic-pulmonary window
		Atrial septal defect associated with abnormalities that cause increased pressure in the left atrium
		Left coronary artery origin from pulmonary artery anomaly
		Continuous murmur at intern mammary artery
		
		Coarctation of the aorta
		Pulmonary atresia
	Extra Precordial	Pulmonary arteriovenous fistula
		Truncus arteriosus
		Anomalies of origin of the pulmonary artery
		A branch of pulmonary artery stenosis
	Venous hum	
EXTRATHORACIC	Cruveilhier-Baumgarten sindrom	
	Sever arterial stenosis	
	Extrathoracic arteriovenos fistulas	

## Continuous precordial murmur

**Patent ductus arteriosus.** A typical continuous murmur can be heard at the patients with patent ductus arteriosus, which is the most frequently described in literature.

It was first described in 1847 in "London Medical Gazette" as "murmur that accompanying first sound...extended to the second sound, so there is no interruption of the murmur before the second sound had already started".

**Figure 2:**
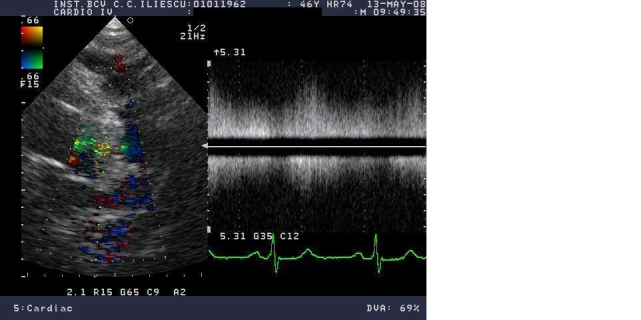
Patent ductus arteriosus. 2D and Doppler transthoracic echocardiography evaluation. In suprasternal section just below the subclavicular artery origin, the continuous flow of patent ductus arteriosus is seen, with maximum velocity of 5,3 m/s.

Later, in 1900, George Gibson presented a more precise description. "It persists trough the second sound and dies away gradually during the long pause. The murmur is rough and trembling. It begins softly and increases in intensity so as to reach its acme just about, or immediately after, the incidence of the second sound, and from that point gradually wanes until its termination” (**Figure 2**) [**2**].

This murmur was also called "a machinery murmur" because of his rough or Gibson's murmur after his name [**2,4**].

Continuous murmurs of patent ductus arteriosus consists of two components: a crescendo systolic one and a decrescendo diastolic one, with a peak of intensity around second sound [**5**]. It is best heard at second left intercostals space or immediately left infraclavicular. It is continuous because the ductus arteriosus provides a permanent communication between the systemic circulation, with high pressure, and the pulmonary circulation, where pressure level is much lower. 

About half of patent ductus arteriosus murmurs in children are not truly continuous and many are only systolic. This is because with the pulmonary vasoconstriction secondary to a large shunt, there is often a moderate degree of pulmonary hypertension, which decreases the aortic–pulmonary artery gradient more in diastole than in systole.

Those with large ductus have been described by Eddy sounds, clicks or crackles at the end of systole and at the beginning of the diastole [**4**].

With the equilibration of aortic and pulmonary artery pressure, systolic flow across the shunt diminishes and finally disappears, leaving the ductus silent (Eisenmenger patent ductus arteriosus). Sometimes, these patients can be recognized by the existence of cyanosis of the lower limbs and left upper limb instead the face, lips and upper right limb are colored as normal (with pulmonary hypertension, unsaturated blood flows through a ductus from the pulmonary artery to the aorta; the ductus often joins the pulmonary artery to the aorta just beyond the left subclavian artery. Unsaturated pulmonary artery blood will then pass beyond the left subclavian artery, and both hands will be less clubbed and cyanotic than the feet. If, however the ductus is at the junction of the aorta and the left subclavian artery, the left hand will be as cyanotic and clubbed as the feet) [**4**]. 

Coronary arteriovenous fistulas. It represents a communication between a coronary artery (most commonly the right coronary) and a cavity of the heart or a large vein that drains at this level [**10**]. Achieving a continuous murmur presupposes the draining in the right cavities, pulmonary artery or coronary sinus (**Figure 4A,B**).

**Figure 4 A:**
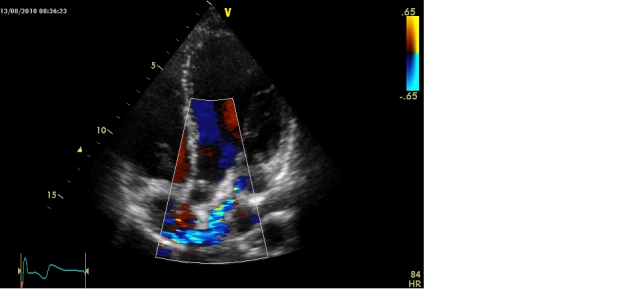
Coronary fistula between coronary left sinus and right atrium.Transthoracic 2D echocardiography - apical 5 chambers –reveal a tortuous ductus that overlaps the image of the left atrium and flows into the right atrium.

**Figure 4 B:**
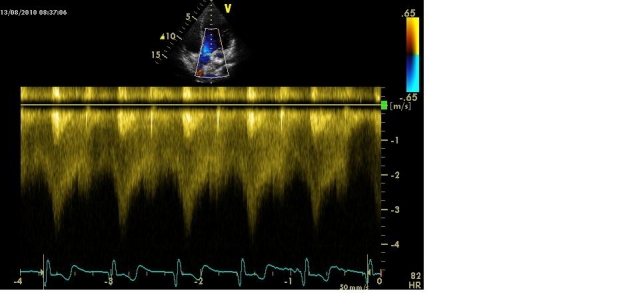
Coronary fistula between coronary left sinus and right atrium. Doppler evaluation at the tortuous ductus (described above) reveals a continuous flow with increased velocity.

**Figure 3:**
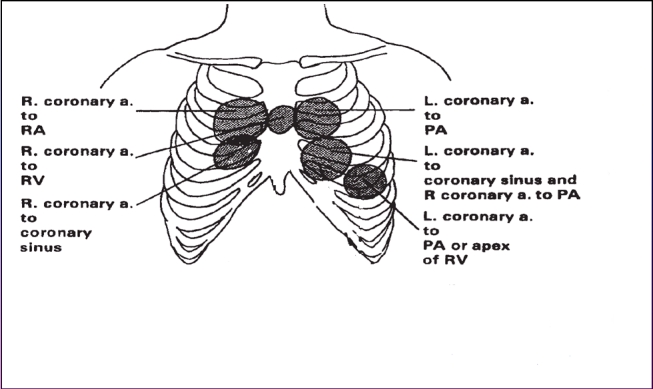
Auscultation sites of coronary fistulas according to place of origin and drainage (modified after [**4**]). Table legend: R-right; L-left; a- artery; RA- right atrium; RV-right ventricle; PA-pulmonary artery

A continuous murmur caused by coronary fistulas is different from that of the patent ductus arteriosus by the place where you can hear it best, usually second intercostals space, left or right parasternal, depending on the artery’s origin and the drain cavity, with maximum of intensity during diastole (**Figure 3**) [**3,4,5,10**]. 

**Sinus of Valsalva aneurysm ruptured into right cavities. ** Aortic sinus aneurysm is most often congenital, but can also occur after an episode of bacterial endocarditis. The most commonly rupture occurs from the right and noncoronary sinuses in to the right atrium or right ventricle. It can cause continuous murmurs that are heard at a maximum at the lower sternal border or xiphoid. Diastolic accentuation of this murmur is an important sign to differentiate ruptured sinus from patent ductus arteriosus or arteriovenous fistula. Systolic suppression of the murmur is caused by both mechanical narrowing of the fistulous tract during systole as well as the probable Venturi effect created by the rapid ejection of blood past the aortic origin of the fistula [**3,4,5,9**].

**Aortic-pulmonary window.**This is a rare congenital disorder in adults, it is a communication between the aorta and pulmonary artery above the semilunar valves, or a secondary disease acquired following aortopulmonare shunts made from surgically produced aortopulmonary palliative connections such as surgeries: Taussing-Blalock, Waterston-Cooley and Potts [**1,3,9**].

Audible continuous murmurs in these conditions can be very difficult to distinguish from those with patent ductus arteriosus. It has the same characteristics but usually is heard on the left of the sternum much lower than a patent ductus arteriosus, which makes it confused with the murmur of ventricular septal defect [**12,13**].

**Atrial septal defect associated with abnormalities that cause increased pressure in the left atrium. **This situation is present in Lutemacher syndrome, when by the interatrial septal defect is realized a continuously left to right shunt, trough increased pressure in the left atrium secondary to mitrale stenosis. Such a continuous murmur is very rare and occurs if the interatrial septum defect is small [**1,4,9**]. 

In cases of anomalous pulmonary venous drainage to the vena cava, right atrium and coronary sinus associated with mitral stenosis or mitral valve atresia , a continuous murmur can also be heard .This continuous murmur can be differentiated by the other continues murmurs at the Valsalva maneuver when its intensity diminishes, in contrast with inspiration that lead to an increase of it [**4,5,12**]. 

**Left coronary artery origin from pulmonary artery anomaly. **This can cause audible precordial continuous murmurs by retrograde flow from the right coronary artery, which comes in the left coronary artery and then flows into the pulmonary artery.

This pathology is very rare and may vary by type ischemic changes occurred electrocardiogram since childhood in left coronary artery territory or infarct in children [**1,2,9**].

**Continuous murmur at intern mammary artery.** Innocent murmurs called often "mammary soufflé" occurs in approximately 10-15% of women at the end of pregnancy and immediately postpartum, this is a continuous murmur with maximum intensity during systole, audible in intercostals spaces II-VI, of the sternal border.

These can be differentiated from the patent ductus arteriosus and coronary fistulae by the existence of a pause between first sound and the beginning of the murmur (the time required to reach the ejection of blood from the left ventricle in to internal mammary artery). Exercise with the stethoscope a light compression to the breast tends to increase its intensity, while an energetic compression with the finger or stethoscope leads to its abolition, also passing standing may diminish its intensity [**2,3,4**].

Continuous murmur of internal mammary artery can be secondary to a fistula between the internal mammary artery and vein, pathology described as rare complication, but increasingly often described in literature, following implantation of a pacemaker or secondary sternal closure after aortic valvuloplasty/valvulotomie. This pathology must be discovered as early as possible, to be interventional solved, because of the increased risk of complications (risk of infection, and further expansion that can lead to heart failure [**9,15,27**]. 

## Continuous extra-precordial murmur

**Coarctation of the aorta. **Continuous murmurs can be heard only in severe coarctation (narrowing of the aortic diameter less than 3 mm) in which the developed collateral circulation is not sufficient to ensure normal flow in diastole, thus makes a continuous gradient systolic-diastolic. The murmur is best heard in the posterior chest, interscapulovertebral, with maximum of intensity in systole.

**Figure 5:**
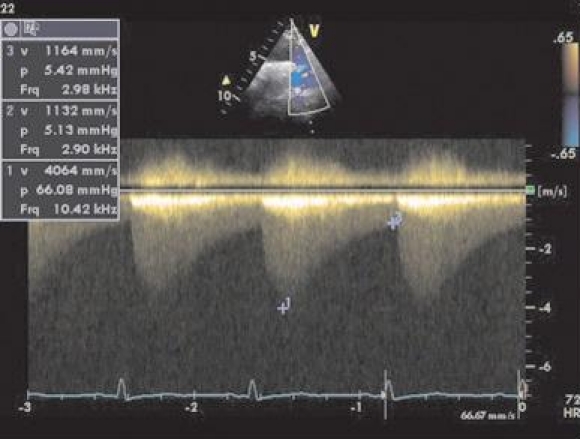
Aortic coarctation. 2D transthoracic echocardiography suprasternal section at patient with sever coarctation of aorta; the Doppler exam reveals a continuous flow with maximum of the velocity of 4,6 m/s.

**Figure 6:**
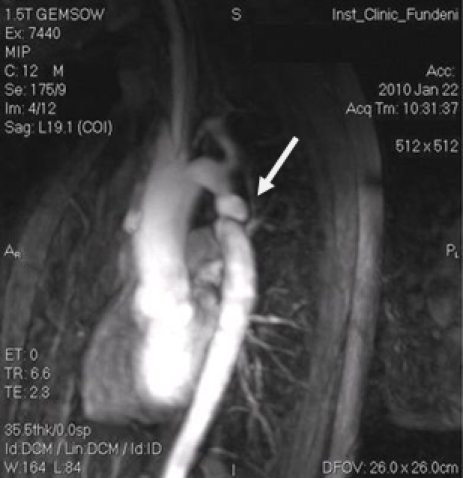
3D angioRM reconstructions at patient with sever aortic coarctation, reveals double aortic isthmus stenosis, with interstenosis ecstasies and aortic arch hypolplasia.

**Figure 7:**
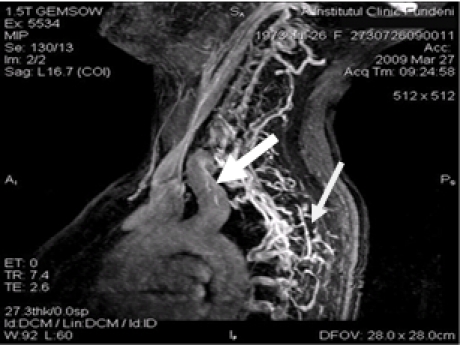
**Arteriovenous fistula between left supraclavicular artery and left vertebral vein. **Head, neck and superior chest magnetic resonance evaluation in a patient with left supraclavicular continuous murmur, with increased cardiac output of 9,4 l/min, showed laterocervical vascular complex malformation. From the dilated left supraclavicular artery origin a lot of tortuous ducts were drained in to left vertebral vein.

In some patients with coarctation of the aorta a continuous murmur may be from the dilated intercostals collateral arteries (**Figure 5**), (**Figure 6**) and (**Figure 7**) [**3,9**].

**Pulmonary artery atresia. **In pulmonary artery atresia, pulmonary circulation is alternate through dilated bronchial collaterals, thus achieving communication between the systemic circulation (bronchial collaterals originate in the aorta) and pulmonary circulation. So it generates a continuous murmur with similar characteristics of the patents ductus arteriosus only that it is widely distributed on the surface of the chest [**3,4,9**].

**Pulmonary arteriovenous fistula.**This can determine a continuous murmur that is superficially heard and usually is associated with cyanosis and typical X-ray changes [**9, 12**]. Patients with pulmonary arterio-venous fistula associated frequently with hereditary hemorrhagic teleangiectazia [**9**].

**Truncus arteriosus. **It is a rare congenital anomaly that can sometimes lead to continuous murmurs, to those with low pulmonary resistance and high pulmonary flow. The diagnosis is suggested by the absence of pulmonary artery to the heart lung radiological examination [**9, 12**].

**Anomalies of origin of the pulmonary artery. **Rarely pulmonary arteries originate from the distal aorta and communicate with pulmonary arterial trunk. It can determinate a continuous murmur by the difference of pressure, in the systemic-pulmonary circulation communication [**9**].

**A branch of pulmonary artery stenosis.** This most often causes a systolic murmur, but the severe one, may extend into the first part of diastole, the murmur is perceived more strongly in the armpit, infraclavicular, interscapulovertebral and more rarely precordial.

It is often associated with other pathologies like: interatrial septum defect, tetralogy of Fallot, pulmonary valve stenosis or ventricular septal defect.

This disease may be caused by pulmonary embolism, lung carcinoma or lung granuloma [**9,23,24**].

## Extra-thoracic continuous murmurs

The venous hum. It was first described in 1867 by Pontain, it is by far the most common physiological continuous murmur, frequently found in healthy children and young. The best heard of the venous hum is just above the clavicle, either medial to the sternocleidomastoid muscle or between its insertions. A venous hum is more likely to be heard on the right side because the right jugular is larger than the left, since it must carry about two-thirds of the intracranial venous drainage .It is more intense in diastole, often harsh and noisy [**2,3**].

Its characteristic is the disappearance of it with jugular vein compressing, or from the transition from sitting to lying position. It can increase at the rotation of the head in the opposite position.

**The venous hum** occurs due to ‘'rapid descent of jugular venous blood mass to the superior vena cava" (P.D. White) 1 this was one of the first hypothesis described, then it was considered that it occurs due to turbulence blood flow in the internal jugular vein vessel deformed by the transverse processes of the atlas during head rotation.

Thyrotoxicosis, anemia and certain cancers, that increase cardiac output may initiate or exacerbate cervical venous hum [**1,2,3,9**]. 

**Cruveilhier-Baumgarten syndrome ("medusa head"). ** Portal venous obstruction, most commonly found in those with cirrhosis, leading to dilated venous collaterals, which determinate so-called "medusa head". The continuous murmur can be heard epigastric and paraombilical [**9**].

**Continuous murmur caused by severs arterial stenosis.** An arterial stenosis (carotid, subclavicular, femoral, renal) greater than 80%, can cause continuous murmurs at the respective site, a continuous murmur with systolic component louder and with short diastolic component. Continuous murmurs in these cases are conditioned by lack of artery collaterals [**3,4,9**].

**Arteriovenous extrathoracic fistulas.** These rare diseases can be congenital or caused by trauma, iatrogenic or may occur even spontaneously. At physical examination the continuous murmur can be detected audibly in systole, accompanied by rustling, and loudest where the fistula is located. The compression of the fistula site abolished murmur, and after stimulation of baroreceptors at this level, it produces sinus bradycardia (sign Branham) and later with it decompression it can produce reflex tachycardia.

The determination of these fistulas is very important because they tend to expand, while achieving a high flux at this level, can lead to the phenomena of congestive heart failure [**8,9**].

**Figure 8:**
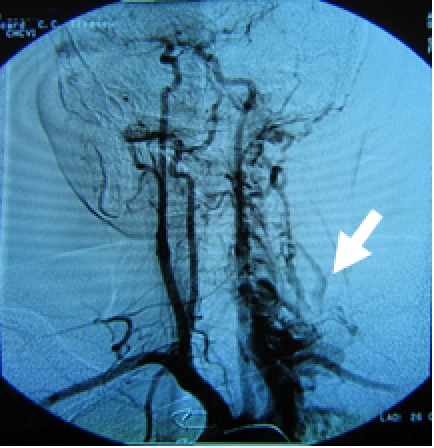
Arteriography head and neck exam, after partial surirgical closure of the malformation described above, reveals the remaining collateral vessels that origine from the first part of the left supraclavicular artery.

It is known that the presence of an arteriovenous fistula larger than 1 cm can lead in about one year to phenomena of cardiac failure (**Figure 7**) and (**Figure 8**). 

Because of this, all the patients with cardiac failure phenomena must be searched for the presence of a continuous murmur anywhere in the body (upper limbs, lower chest, head, chest and neck).

**Cardiovascular auscultation remains one of the most important, simple and inexpensive clinical investigation, which, in those with continuous murmurs can lead to a possible diagnosis and establish a targeted strategy for evaluation and treatment.**
